# The safety and efficacy of clevidipine for blood pressure management in neurocritical patients: a systematic review and meta-analysis

**DOI:** 10.1038/s41598-024-54667-9

**Published:** 2024-03-16

**Authors:** Monika Widiastuti, Dewi Yulianti Bisri, Iwan Abdul Rachman

**Affiliations:** 1 Department of Anesthesiology, Universitas Padjajaran, Bandung, West Java Indonesia; 2 Department of Anesthesiology, Faculty of Medicine, Universitas Pelita Harapan, Tangerang, Banten Indonesia; 3 Department of Anesthesiology, Siloam Hospital Lippo Village, Tangerang, Banten Indonesia

**Keywords:** Clevidipine, Blood pressure, Hypertension, Neuro critical patients, Drug therapy, Therapeutics

## Abstract

We aim to determine the safety and efficacy of clevidipine for neurocritical patients. To comprehensively identify relevant studies, a systematic search strategy was employed using the following keywords: “clevidipine”, “high blood pressure”, “hypertension”, “Neuroscience Intensive Care”, “neuro critical”, and “neurosurgical patients”. Searches were conducted in the Clinicaltrials.gov, PubMed, and EuroPMC databases, with the search extending until September 1, 2023. The primary outcomes of interest were the time needed to achieve the target systolic blood pressure (SBP) and the percentage of time a patient remained within the targeted SBP range. Secondary outcomes included SBP values, duration of intensive care unit (ICU) stay in days, rates of hypotension, and rates of tachycardia. We included five retrospective cohort studies (n = 443), utilizing nicardipine as the primary comparator. Comparison of the time to reach target systolic blood pressure (SBP) revealed no significant difference between medications (SMD = − 1.09, p = 0.33). Likewise, the achieved SBP target showed no notable distinction (RR = 1.15, p = 0.81). However, clevidipine exhibited a slightly higher percentage of time within the target SBP range (SMD = 0.33, p = 0.04), albeit with moderate heterogeneity. Importantly, all included studies were retrospective cohort studies, underscoring the methodological context of the investigation. Clevidipine and the control group were found to be comparable in terms of achieving target SBP. Clevidipine may have a slight advantage in maintaining blood pressure within the desired range, but further research is needed to confirm this finding.

## Introduction

Acute blood pressure reduction is a critical aspect of managing patients in the neuro intensive care unit (ICU)^1^. Firstly, elevated blood pressure can exacerbate neurological damage and increase the risk of complications in patients with acute neurological conditions such as intracerebral hemorrhage. Uncontrolled hypertension in the neuro ICU can lead to increased intracranial pressure (ICP) and impaired cerebral perfusion^1,2^. Elevated blood pressure can contribute to increased ICP, which can further compromise cerebral blood flow and oxygen delivery to the brain. Managing blood pressure in the neuro ICU requires a delicate balance between reducing blood pressure to prevent further damage and maintaining adequate cerebral perfusion^1^. Therefore, it is essential to carefully monitor and control blood pressure in these patients to optimize outcomes.

One potential treatment option for this purpose is clevidipine, a calcium channel blocker, and its usage has been investigated in neurosurgical patients. Studies have demonstrated the efficacy of clevidipine in rapidly lowering blood pressure, with a median time to target systolic blood pressure reduction of 5.3–6.0 min^3,4^. The safety profile has been assessed and found that lower infusion rates of clevidipine achieved the desired blood pressure control without dose-related adverse reactions. Another study reported on the use of clevidipine in pediatric patients, including its use for perioperative hypertension control and controlled hypotension during orthopedic surgical procedures^5^.

In addition to its antihypertensive effects, clevidipine has been found to have other potential benefits. One study suggested that clevidipine administration was not associated with a reflex increase in heart rate or change in cardiac index, making it a suitable option for patients who have received beta-blocking agents or are atrially paced^6^. Another study explored the mechanisms of clevidipine action and found that it provided superior dyspnea relief compared to standard intravenous antihypertensives in patients with hypertensive acute heart failure^7^.

Studies have primarily focused on the use of clevidipine in cardiac surgery and in patients with acute severe hypertension in intensive care units and emergency departments^8^. Little is known about the application of clevidipine for neurocritical patients even though there are several evidence to suggest that clevidipine may also have benefits in neurocritical care. Hence, the objective of current study is to determine the safety and efficacy of clevidipine for neurocritical patients.

## Methods

To comprehensively identify relevant studies, a systematic search strategy was employed using the following keywords: “clevidipine”, “high blood pressure”, “hypertension”, “Neuroscience Intensive Care”, “neuro critical”, and “neurosurgical patients”. Searches were conducted in the Clinicaltrials.gov, PubMed, and EuroPMC databases, with the search extending until September 1, 2023 (Table 1). No language restrictions were applied, and preprints were considered eligible for inclusion. The inclusion criteria encompassed all types of manuscripts that reported outcomes of interest. Exclusion criteria included grey literature, abstract-only publications, letters to the editor, and other equivalent formats. Animal studies were excluded from the analysis.

**Table 1:** Search strategy for current systematic review and meta-analysis.

Search queries	Database
("clevidipine"[Supplementary Concept] OR "clevidipine"[All Fields]) AND ("hypertension"[MeSH Terms] OR "hypertension"[All Fields] OR ("high"[All Fields] AND "blood"[All Fields] AND "pressure"[All Fields]) OR "high blood pressure"[All Fields] OR ("hypertense"[All Fields] OR "hypertension"[MeSH Terms] OR "hypertension"[All Fields] OR "hypertension s"[All Fields] OR "hypertensions"[All Fields] OR "hypertensive"[All Fields] OR "hypertensive s"[All Fields] OR "hypertensives"[All Fields])) AND ((("neuroscience s"[All Fields] OR "neurosciences"[MeSH Terms] OR "neurosciences"[All Fields] OR "neuroscience"[All Fields]) AND ("critical care"[MeSH Terms] OR ("critical"[All Fields] AND "care"[All Fields]) OR "critical care"[All Fields] OR ("intensive"[All Fields] AND "care"[All Fields]) OR "intensive care"[All Fields])) OR (("neurology"[MeSH Terms] OR "neurology"[All Fields] OR "neuro"[All Fields] OR "neuros"[All Fields]) AND ("critical"[All Fields] OR "critically"[All Fields])) OR (("neurosurgic"[All Fields] OR "neurosurgical"[All Fields] OR "neurosurgically"[All Fields]) AND ("patient s"[All Fields] OR "patients"[MeSH Terms] OR "patients"[All Fields] OR "patient"[All Fields] OR "patients s"[All Fields])))	EuroPMC
Clevidipine AND High blood pressure OR hypertension AND Neuroscience Intensive Care OR neuro critical OR neurosurgical patients	PubMed
Clevidipine AND High blood pressure OR hypertension AND Neuroscience Intensive Care OR neuro critical OR neurosurgical patients	Clinicaltrials.gov

In the conducted systematic review and meta-analysis, the study population comprised neurocritical patients. Neurocritical patients were defined as individuals who suffered from intracerebral hemorrhage, subarachnoid hemorrhage, and ischemic stroke. The investigated intervention involved the administration of clevidipine. The control cohort consisted of individuals who did not receive clevidipine, potentially receiving interventions such as saline, nicardipine, placebo, or no pharmacological intervention. The primary outcome of interests was on the systolic blood pressure following clevidipine administration and examining both the total and percentage of time required to attain the specified target SBP. The target SBP was defined as being below 140 mmHg for intracerebral hemorrhage, below 160 mmHg for subarachnoid hemorrhage, and below 180 mmHg for ischemic stroke. Secondary outcomes, such as the duration of ICU stay, total occurrences of tachycardia, and total instances of hypotension, were also considered.

The screening process of studies extracted from each database involved several steps. Duplicates were first removed using the automated deduplication feature provided by rayyan.ai. Eligible studies were then screened by title and abstract for relevance. Those selected for full-text review were assessed against predetermined inclusion and exclusion criteria. To ensure rigor and reliability, all authors participated in both stages of screening, and conflicts were resolved through discussion to achieve consensus. Articles selected for final inclusion were subjected to a detailed data extraction process. This included the assessment of bibliographic data, study design, participant information (including dosing details, duration of administration, control group characteristics, and diagnoses of patients admitted to the ICU), and intervention details related to clevidipine (such as regimen details and adverse reactions). Primary and secondary outcome data were also extracted.

Quality assessment of eligible studies was performed independently by all authors using the Newcastle Ottawa Scale (NOS) for cohort studies. The NOS assesses the quality of non-randomized studies by evaluating selection, comparability, and outcome. It assigns a score based on predefined criteria, with higher scores indicating higher study quality. This scale was utilized to gauge the methodological quality of the included cohort studies in the systematic review. When there were disagreements of quality assessment of eligible studies, all authors resolved the disagreements through online discussion.

The data was analyzed using R software version 3.5.3 and Rstudio version 1.2.5003. To aggregate continuous variables, a method based on inverse variance was employed to calculate the mean differences and their corresponding standard deviations. The Maentel-Haenszel formula was utilized for computing dichotomous variables to derive risk ratios together with 95% confidence intervals. Random-effects models were applied for conducting pooled analysis regardless of heterogeneity. All statistical tests were two-tailed, and significance level was set at ≤ 0.05.

## Results

**Figure 1:**
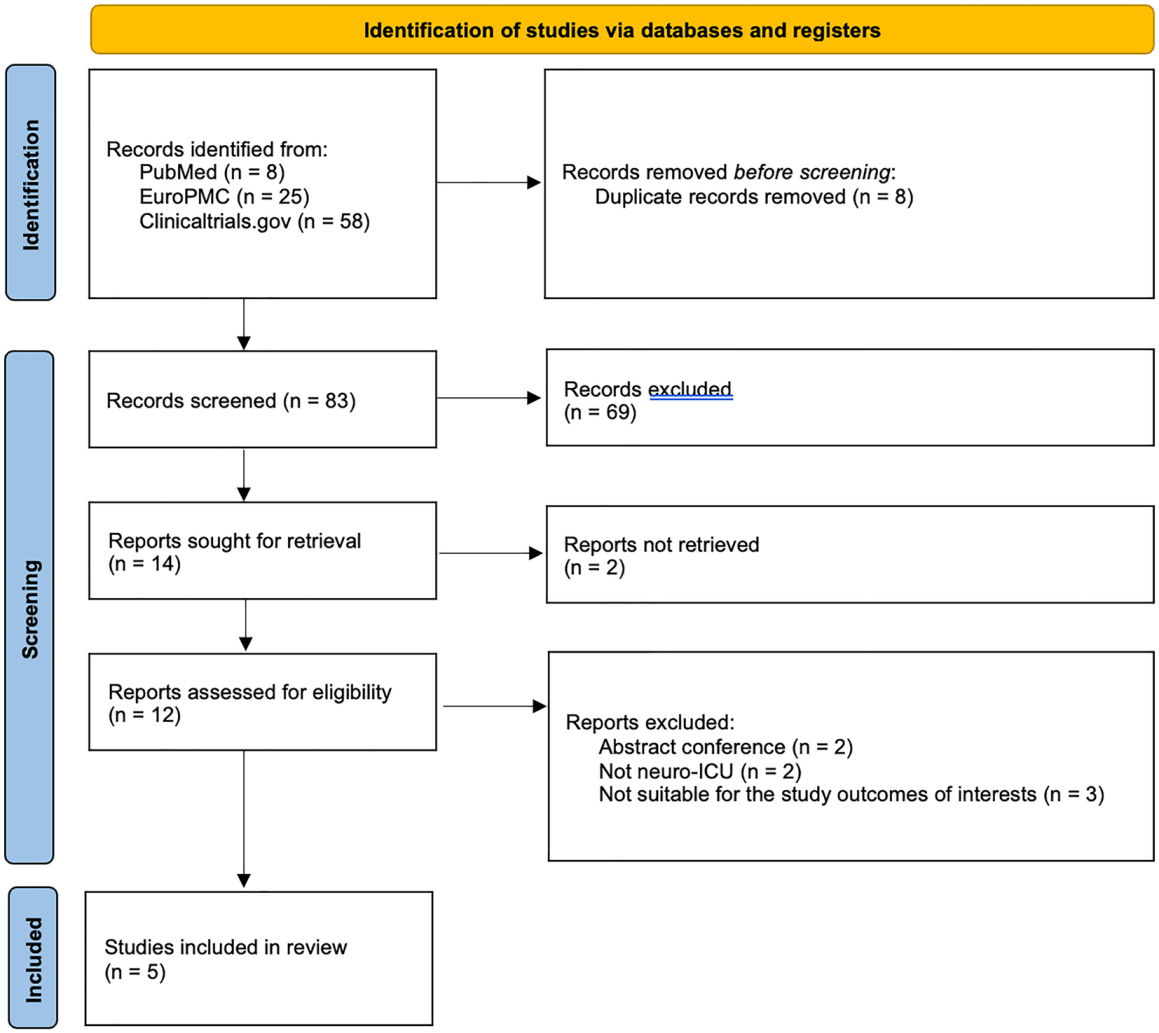
PRISMA flow diagram.

We followed the PRISMA guidelines to conduct a comprehensive literature search and selection process for this review. A total of 91 articles were retrieved from electronic databases such as PubMed, EuroPMC, and Clinicaltrials.gov. After evaluating the abstracts, 69 articles were excluded as they did not pertain to the use of clevidipine for blood pressure management among neurocritical patients. From the remaining pool, 12 full-text articles underwent further assessment based on eligibility criteria resulting in seven more publications being excluded that did not meet inclusion or exclusion criteria. Ultimately, our review included five retrospective studies which are outlined in Fig. 1 providing detailed PRISMA flow information^9–13^.

Clevidipine dosing varies across different studies and contexts. Borrell-Vega in 2020 used an initial dose of 10.8 mg/h with a 9.1 mg/h increase^11^. Finger in 2016 employed a range from 1.5 to 8 mg/h with a median of 3 mg/h^10^. Rosenfeldt in 2018 administered a fixed dose of 2 mg/h. Rodriguez in 2022 utilized an average dose of 1 mg/h^12^. Meanwhile, Allison in 2017 started at 2 mg/h and doubled the dose every 90 s until reaching a rate of 12 mg/h, then increased it in 4 mg/h increments up to a maximum dose of 32 mg/h^9^. In these studies about neurocritical care, patients were diagnosed with various conditions: ICH, AIS, SAH, SDH, and others For ICH, the number of patients in each study ranged from as low as 3 to as high as 144. In the case of acute ischemic stroke, the numbers ranged from 1 to 77 patients across the studies. 12 patients with ASH, 73 patients with SDH and an additional 59 patients falling into various other diagnostic categories. It is noteworthy that nicardipine served as the primary comparator to clevidipine in all studies that were included. We also noted various definition of hypotension and tachycardia among included studies. Detailed characteristics of included studies were presented in Tables 2 and 3.

**Table 2:** Demographic characteristics of included studies for the management of hypertension in neurocritical patients.

Study ID, NOS	Study design	Clev details	Control details	Total cohort		Diagnosis					Target BP	Hypo tension	Tachy cardia
				Clev	Control	ICH	AIS	SAH	SDH	Others			
Borrell-Vega 2020, 7	Retro	10.8 + 9.1 mg/h	Nicardipine; max. 15 mg/h	12	12	7	1	3	–	1	SBP < 140 mm Hg for ICH, < 160 mm Hg for SAH and < 180 mm Hg for IS	SBP < 90 mmHg	> 100 bpm
Finger 2016, 7	Retro	3 (1.5–8) mg/h	Nicardipine, 5 (4–7) mg/h	19	38	24	6	–	–	27	Not defined	SBP < 100 mmHg	> 120 bpm
Rosenfeldt 2018, 8	Retro	2 mg/h	Nicardipine; n.r	59	60	37	1	5	73	20	Not defined	SBP < 100 mmHg	> 120 bpm
Rodriguez 2022, 8	Retro	1 ± 1 mg/h	None	33	–	3	6	4	–	11	SBP maintained for more than 75% of infusion length and no need of rescue treatment with different intravenous antihypertensive drugs	SBP < 80 mmHg for at least 5 min within 1 h of infusion begin	Not defined
Allison 2017, 7	Retro	2 mg/h; doubling the dose every 90 s until a rate of 12 mg/h was reached, and then increasing by 4 mg/h increments to a maximum dose of 32 mg/h	Nicardipine; initially 5 mg/h titrated per 15 min to a maximum of 15 mg/h	70	140	133	77	–	–	–	SBP < 180 mmHg for AIS or < 150 mmHg for ICH	SBP < 91 mmHg	Not defined

*AIS* acute ischemic stroke, *bpm* beats per minute, *BP* blood pressure, *Clev* clevidipine, *h* hour, *ICH* intracerebral hemorrhage, *retro* retrospective, *SAH* subarachnoid hemorrhage, *SBP* systolic blood pressure, *SDH* Subdural hematoma, *mg* milligram, *mmHg* millimeter of mercury, *NOS* Newcastle Ottawa Scale.

**Table 3:** Primary endpoint comparison within treatment groups for the management of hypertension in neurocritical patients.

Study ID	Time to achieve target SBP, hours		Percentage of time in target SBP range, %		Achieved systolic blood pressure goal, n	
	Clev	Control	Clev	Control	Clev	Control
Borrell-Vega 2020	29.6 ± 11.1	52.1 ± 17.7	90.8 ± 8.3	74.7 ± 12.9	n.r	n.r
Finger 2016	34 ± 12	52.6 ± 22.5	79.2 ± 6.8	77.9 ± 7.3	8	15
Rosenfeldt 2018	n.r	n.r	65.8 ± 101	65.5 ± 108.2	30	26
Rodriguez 2022	71 ± 48	N/A	64 ± 89	N/A	75 out of 103 patients, no control group	
Allison 2017	Reported in 2- and 24-h period		80 ± 19	75 ± 19	AIS = 12 vs 16 patients; ICH: 21 vs 17 patients	

*Clev* clevidipine, *n* number, *N/A* not applicable, *n.r* not reported, *SBP* systolic blood pressure.

In this meta-analysis comparing clevidipine and nicardipine, several key outcome measures were evaluated. First, the time to achieve the target SBP did not significantly differ between the two medications, with a standardized mean difference (SMD) of − 1.09 favoring clevidipine, but with no statistical significance (p = 0.33). Similarly, the achieved SBP target showed no substantial difference between the two drugs, with a relative risk (RR) of 1.15 (p = 0.81). However, the percentage of time spent within the target SBP range was slightly higher for clevidipine, as indicated by an SMD of 0.33 (p = 0.04), albeit with moderate heterogeneity.

**Figure 2:**
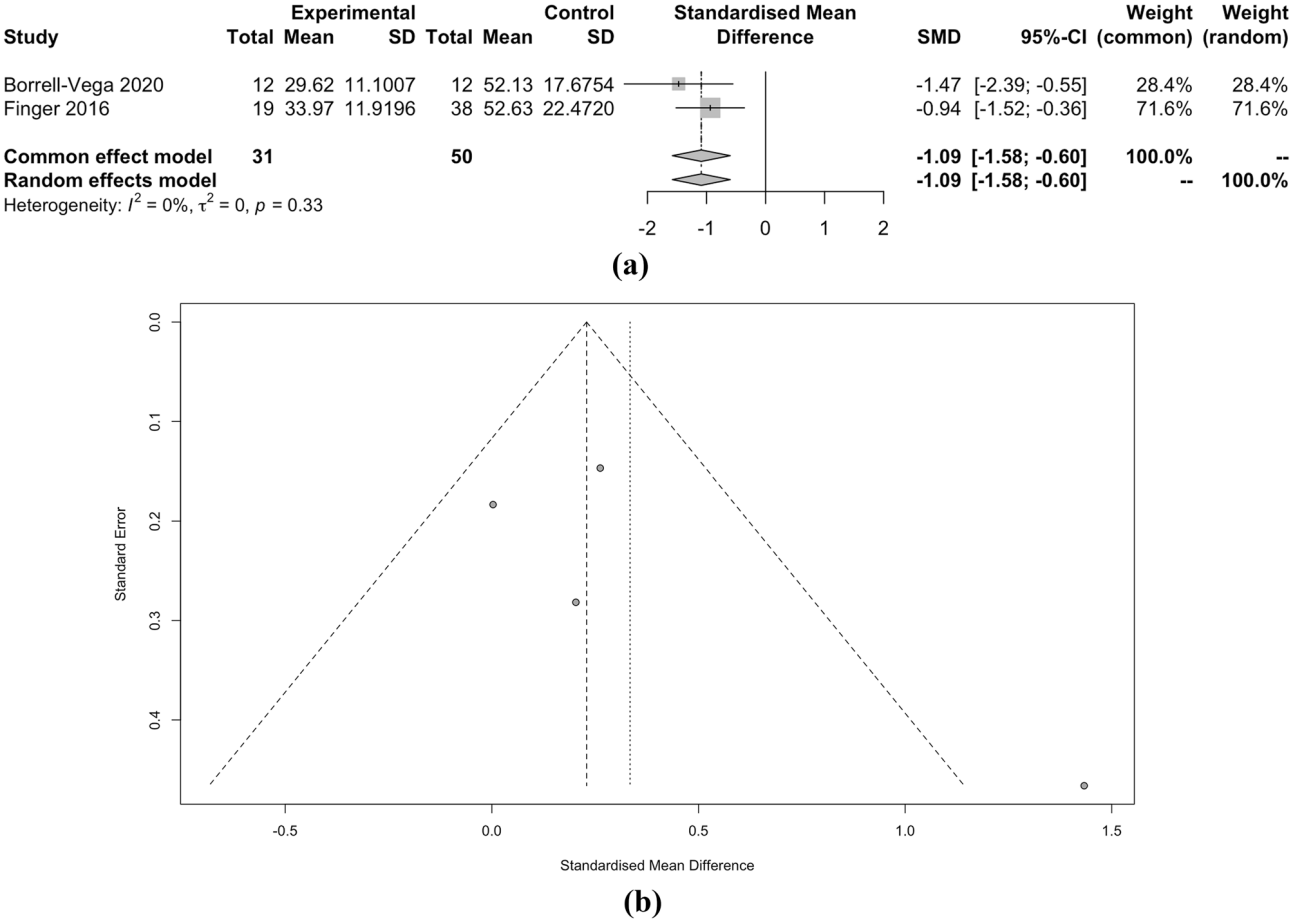
(**a**) Forest plot and (**b**) funnel plot of total time to achieve target SBP.

**Figure 3:**
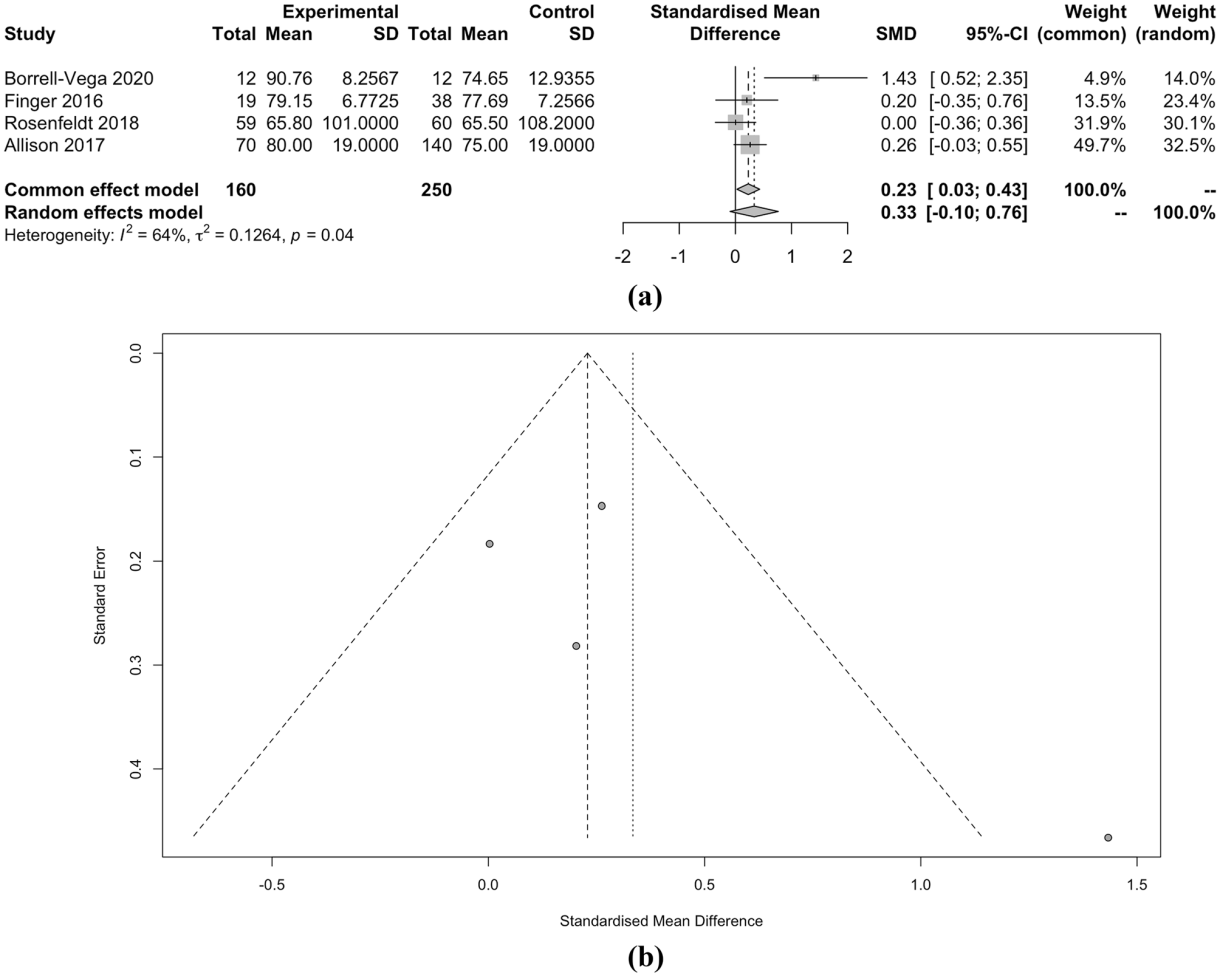
(**a**) Forest plot and (**b**) funnel plot of percentage of time in target SBP.

**Figure 4:**
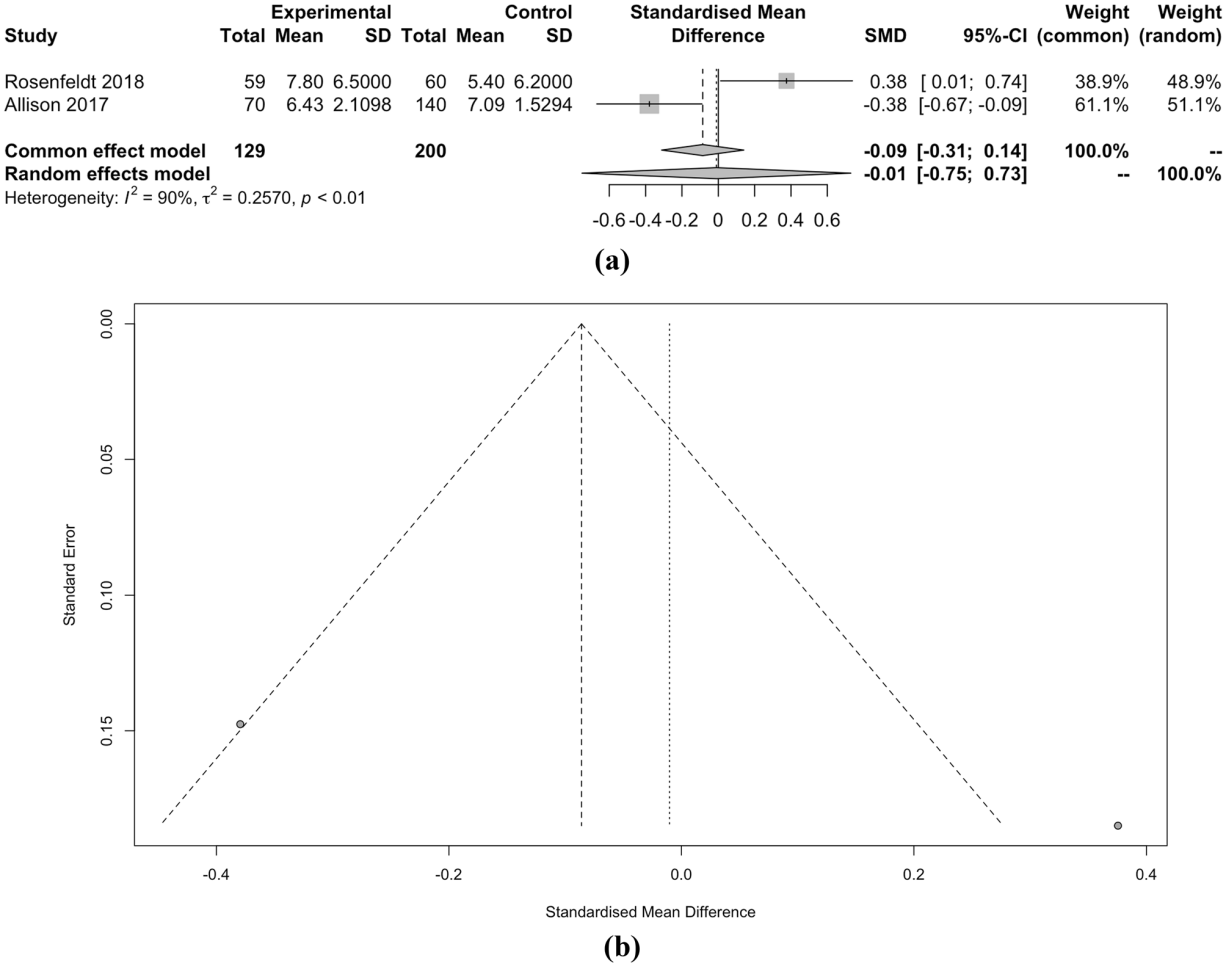
(**a**) Forest plot and (**b**) funnel plot of ICU stay.

**Figure 5:**
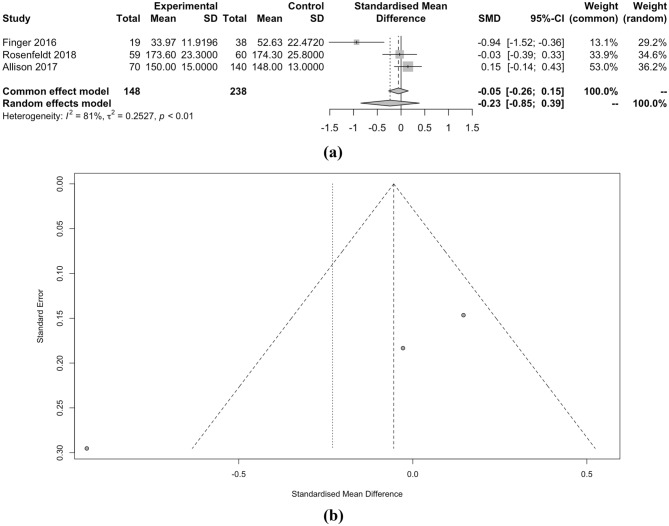
(**a**) Forest plot and (**b**) funnel plot of post-clevidipine administration SBP.

**Figure 6:**
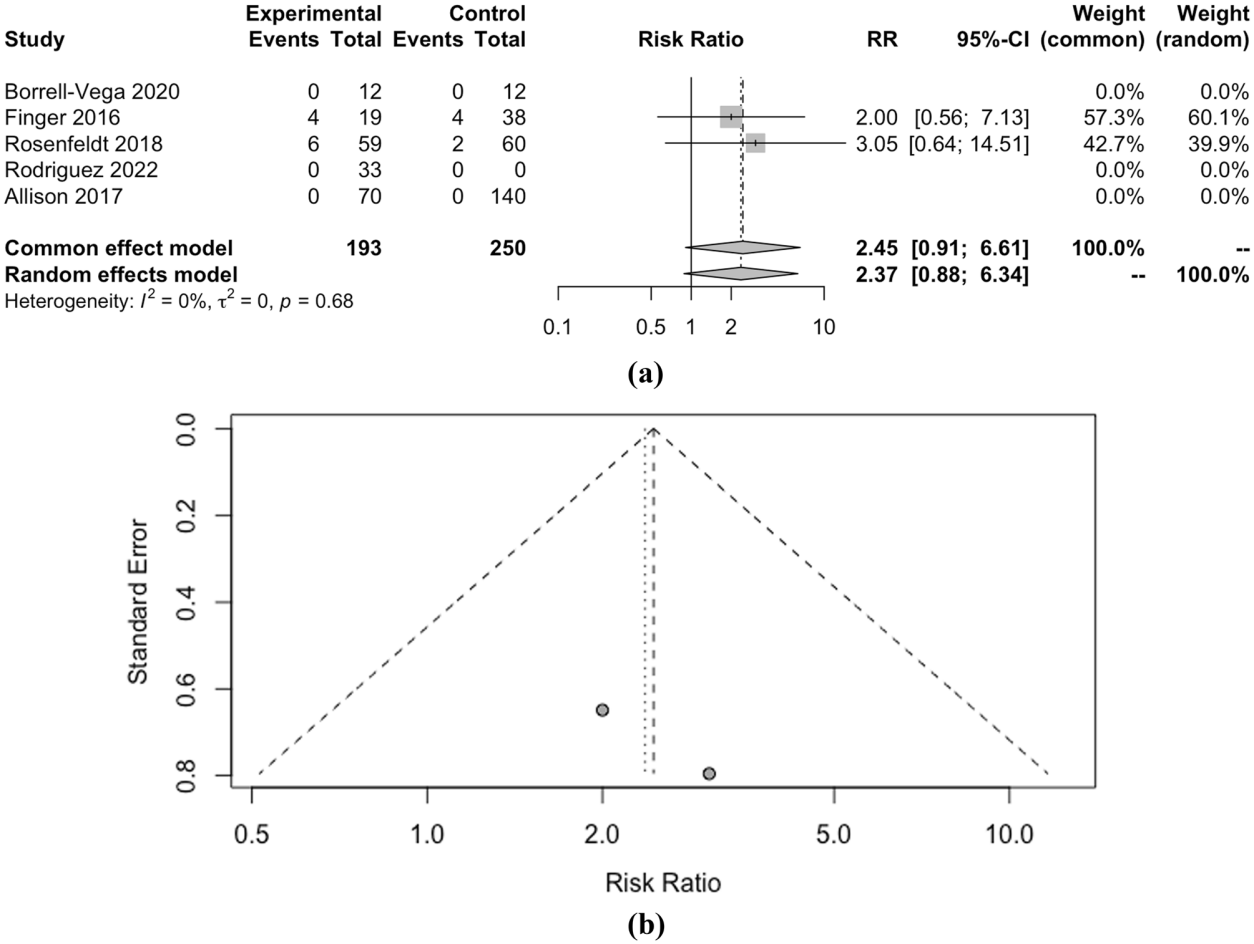
(**a**) Forest plot and (**b**) funnel plot of total events tachycardia.

**Figure 7:**
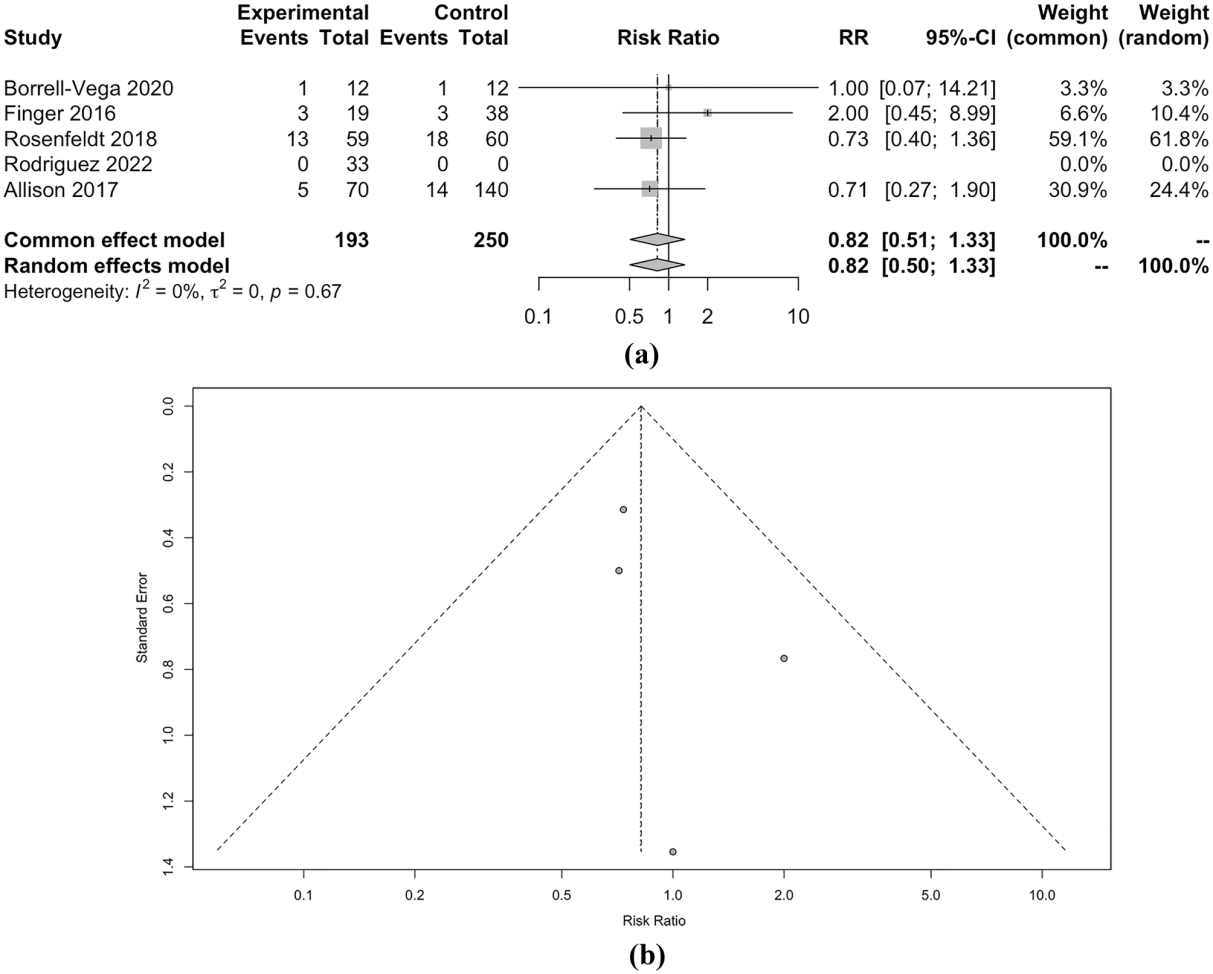
(**a**) Forest plot and (**b**) funnel plot of total events hypotension.

When considering SBP as a continuous measure, clevidipine demonstrated a marginally lower SMD of − 0.23 compared to nicardipine, but this difference lacked statistical significance (p < 0.01). Moreover, the length of ICU stay and the occurrence of hypotension did not significantly differ between the two drugs, with SMD of − 0.01 and RR of 0.82, respectively (both p < 0.01). However, the incidence of tachycardia, as indicated by RR of 2.37, also showed no significant distinction between clevidipine and nicardipine (p = 0.68). Detailed meta-analysis can be seen in Figs. 2, 3, 4, 5, 6 and 7.

## Discussion

Our meta-analysis suggests that both clevidipine and control group are comparable in terms of achieving target SBP, though clevidipine may have a slight advantage in maintaining blood pressure within the desired range, albeit with some heterogeneity in the data. Additionally, they exhibit similar effects on ICU stay duration, hypotension, and tachycardia. This finding is consistent with previous studies such as the ECLIPSE trials and the ESCAPE-2 trial, which demonstrated the efficacy of clevidipine in treating acute hypertension in cardiac surgery patients^14,15^.

The finding that clevidipine demonstrated a slight advantage in maintaining blood pressure within the desired range is of particular clinical significance. In critical care scenarios, maintaining stable blood pressure is crucial to prevent complications such as cerebral ischemia or hemorrhage, myocardial infarction, and organ damage^16,17^. Clevidipine, as a calcium channel blocker, acts by dilating peripheral arteries, reducing vascular resistance, and subsequently lowering blood pressure^6^. This mechanism may contribute to its ability to provide more consistent control of blood pressure within the target range. However, it is essential to address the observed heterogeneity in the data. This heterogeneity could arise from several factors, including variations in patient characteristics, comorbidities, dosing regimens, and the specific clinical contexts in which clevidipine was administered across the included studies. These discrepancies highlight the importance of considering patient-specific factors and clinical nuances when deciding on the most suitable blood pressure management strategy. It is important to mention that nicardipine was used as the main point of comparison against clevidipine in all the studies that were analyzed. Furthermore, future research should aim to identify the specific patient populations or scenarios in which the advantage of clevidipine in blood pressure control is most pronounced, allowing for more tailored and effective treatment decisions.

Additionally, our meta-analysis revealed that both clevidipine and the control group had similar effects on other important clinical outcomes, including ICU stay duration, hypotension, and tachycardia. These findings suggest that, aside from the advantage in blood pressure control, clevidipine does not significantly differ from the control group in terms of these crucial parameters. Clevidipine and the control group had similar effects on ICU stay duration, hypotension, and tachycardia. This means that clevidipine does not confer any additional benefits in terms of these outcomes compared to the control group. These findings are in line with previous research on the topic. For example, a study by Polderman et al. demonstrated that the use of thiopental, a sedative agent, can induce circulatory depression in a dose-dependent manner, which may lead to hypotension and tachycardia^18^.

It is essential to acknowledge several inherent limitations that may influence the interpretation of the results. Firstly, the analysis found no significant difference in the time to achieve the target SBP between the two medications. However, the limited number of studies included, and the associated sample sizes may impact the statistical power to detect a true difference^19^. Secondly, the achieved SBP target did not significantly differ between clevidipine and nicardipine, but the confidence interval suggests potential variability in the effect estimate, and the relatively small number of studies could limit the precision of this finding. Thirdly, the percentage of time spent within the target SBP range showed some heterogeneity among studies, which may introduce uncertainty into the observed effect. Additionally, the heterogeneity observed in the analysis of SBP as a continuous measure, along with the significant variability in clevidipine dosing strategies across studies, could affect the accuracy of the overall effect estimate. Furthermore, the high heterogeneity in the analysis of ICU stays duration and the occurrence of hypotension raises questions about the consistency of these outcomes across the included studies. Lastly, while the analysis found no significant difference in the incidence of tachycardia between clevidipine and nicardipine, the relatively wide confidence interval suggests that the true effect could fall within a broad range.

The findings of this meta-analysis hold important clinical implications for blood pressure management in various healthcare settings. Firstly, the analysis indicates that clevidipine and nicardipine demonstrate similar efficacy in achieving the target systolic blood pressure. This implies that clinicians have the flexibility to choose between these two intravenous antihypertensive agents, considering factors like patient preferences, medication availability, and cost-effectiveness. Secondly, the observed advantage of clevidipine in maintaining blood pressure stability within the desired range highlights its potential suitability for critical care scenarios where precise blood pressure control is critical^20–22^. Moreover, the comparable safety profiles of both medications in terms of adverse events like hypotension and tachycardia provide reassurance to clinicians, indicating that neither medication significantly elevates the risk of these side effects^23^. Nonetheless, the decision between clevidipine and nicardipine should be tailored to individual patient factors, such as comorbidities and the specific clinical circumstances. Lastly, the identified heterogeneity in some outcomes and the inherent limitations of the included studies underscore the need for further research in this area. Future investigations involving larger and more diverse patient populations, along with standardized dosing regimens, can enhance our understanding of the comparative effectiveness of these medications in blood pressure management, facilitating more informed clinical decision-making.

Clevidipine and the control group were found to be comparable in terms of achieving target SBP. Clevidipine may have a slight advantage in maintaining blood pressure within the desired range, but further research is needed to confirm this finding. Both clevidipine and the control group had similar effects on ICU stay duration, hypotension, and tachycardia. However, the presence of heterogeneity in the data suggests that caution should be exercised when interpreting these results. Future studies should aim to address the limitations of the included studies and provide more robust evidence on the efficacy and safety of clevidipine compared to the control group.

## Data Availability

Available upon reasonable requests by contacting the corresponding author.

## Abbreviations

AIS: Acute ischemic stroke
CI: Confidence interval
ICH: Intracerebral hermorrhage
ICU: Intensive care unit
ICP: Intracranial pressure
PRISMA: Preferred reporting items for systematic reviews and meta-analyses
PROSPERO: International prospective register of systematic reviews
RR: Risk ratio
SAH: Subarachnoid hemorrhage
SDH: Subdural hemorrhage
SBP: Systolic blood pressure
SMD: Standard mean difference

## References

[1] Der-Nigoghossian C, Levasseur-Franklin K, Makii J (2019). Acute blood pressure management in neurocritically ill patients. Pharmacotherapy.

[2] Anderson CS (2010). Effects of early intensive blood pressure-lowering treatment on the growth of hematoma and perihematomal edema in acute intracerebral hemorrhage: The intensive blood pressure reduction in acute cerebral haemorrhage trial (INTERACT). Stroke.

[3] Cobb A, Thornton L (2018). Sodium nitroprusside as a hyperinflation drug and therapeutic alternatives. J. Pharm. Pract..

[4] Espinosa A (2016). Perioperative use of clevidipine: A systematic review and meta-analysis. PLoS ONE.

[5] Towe E, Tobias JD (2010). Preliminary experience with clevidipine in the pediatric population. J. Intensive Care Med..

[6] Gradman AH, Vivas Y (2007). New therapeutic perspectives with clevidipine: An ultra-short-acting intravenous Ca^2+^ channel blocker. Expert Opin. Investig. Drugs.

[7] Dahl GP (2016). High affinity complexes of pannexin channels and L-type calcium channel splice-variants in human lung: Possible role in clevidipine-induced dyspnea relief in acute heart failure. EBioMedicine.

[8] Erickson AL, DeGrado JR, Fanikos JR (2010). Clevidipine: A short-acting intravenous dihydropyridine calcium channel blocker for the management of hypertension. Pharmacotherapy.

[9] Allison TA (2019). Comparison of clevidipine and nicardipine for acute blood pressure reduction in patients with stroke. J. Intensive Care Med..

[10] Finger JR, Kurczewski LM, Brophy GM (2017). Clevidipine versus nicardipine for acute blood pressure reduction in a neuroscience intensive care population. Neurocrit. Care.

[11] Borrell-Vega J, Uribe AA, Palettas M, Bergese SD (2020). Clevidipine use after first-line treatment failure for perioperative hypertension in neurosurgical patients: A single-center experience. Medicine.

[12] Rosenfeldt Z (2018). Comparison of nicardipine with clevidipine in the management of hypertension in acute cerebrovascular diseases. J. Stroke Cerebrovasc. Dis..

[13] Rodriguez, B. E., Arana-Arri, E., Boedo, M. J. M. & Ruiz, A. M. *Perioperative Control of Acute High Blood Pressure in Neurosurgical Patients Admitted to Intensive Care Unit Using Clevidipine (Neuro-Clev)*. (2022) 10.21203/rs.3.rs-1422741/v1.

[14] Aronson S (2008). The ECLIPSE trials: Comparative studies of clevidipine to nitroglycerin, sodium nitroprusside, and nicardipine for acute hypertension treatment in cardiac surgery patients. Anesth. Analg..

[15] Singla N (2008). Treatment of acute postoperative hypertension in cardiac surgery patients: an efficacy study of clevidipine assessing its postoperative antihypertensive effect in cardiac surgery-2 (ESCAPE-2), a randomized, double-blind, placebo-controlled trial. Anesth. Analg..

[16] Seifi A (2023). Comparison between clevidipine and nicardipine in cerebrovascular diseases: A systematic review and meta-analysis. Clin. Neurol. Neurosurg..

[17] Saldana S (2022). Comparison of clevidipine and nicardipine for acute blood pressure reduction in hemorrhagic stroke. Neurocrit. Care.

[18] Polderman KH, Tjong Tjin Joe R, Peerdeman SM, Vandertop WP, Girbes ARJ (2002). Effects of therapeutic hypothermia on intracranial pressure and outcome in patients with severe head injury. Intensive Care Med..

[19] Lee YH (2018). An overview of meta-analysis for clinicians. Korean J. Intern. Med..

[20] Brown CS (2022). Comparison of intravenous antihypertensives on blood pressure control in acute neurovascular emergencies: A systematic review. Neurocrit. Care.

[21] Kamp A (2022). Comparison of intermittent versus continuous infusion antihypertensives in acute ischemic stroke. Am. J. Emerg. Med..

[22] De Gaudio AR, Chelazzi C, Villa G, Cavaliere F (2009). Acute severe arterial hypertension: Therapeutic options. Curr. Drug Targets.

[23] Hariri L, Patel JB (2023). Vasodilators. StatPearls.

